# Correlation between nonalcoholic fatty liver disease and left ventricular diastolic dysfunction in non-obese adults: a cross-sectional study

**DOI:** 10.1186/s12876-023-02708-4

**Published:** 2023-03-27

**Authors:** Fangyuan Cong, Luying Zhu, Lihua Deng, Qian Xue, Jingtong Wang

**Affiliations:** Geriatric Department, Peking University People’s Hospital, Beijing, 100044 China

**Keywords:** Non-obese subjects, Nonalcoholic fatty liver disease, Left ventricle, Ventricular dysfunction

## Abstract

**Background and aims:**

Non-alcoholic fatty liver disease (NAFLD) is associated with a greater risk of developing cardiovascular disease and have adverse impacts on the cardiac structure and function. Little is known about the effect of non-obese NAFLD upon cardiac function. We aimed to compare the echocardiographic parameters of left ventricle (LV) between non-obese NAFLD group and control group, and explore the correlation of non-obese NAFLD with LV diastolic dysfunction.

**Methods and results:**

In this cross-sectional study, 316 non-obese inpatients were enrolled, including 72 participants with NAFLD (non-obese NAFLD group) and 244 participants without NAFLD (control group). LV structural and functional indices of two groups were comparatively analyzed. LV diastolic disfunction was diagnosed and graded using the ratio of the peak velocity of the early filling (E) wave to the atrial contraction (A) wave and E value. Compared with control group, the non-obese NAFLD group had the lower E/A〔(0.80 ± 0.22) vs (0.88 ± 0.35), t = 2.528, *p* = 0.012〕and the smaller LV end-diastolic diameter〔(4.51 ± 0.42)cm vs (4.64 ± 0.43)cm, t = 2.182, *p* = 0.030〕. And the non-obese NAFLD group had a higher prevalence of E/A < 1 than control group (83.3% vs 68.9%, X^2^ = 5.802, *p* = 0.016) while two groups had similar proportions of LV diastolic dysfunction (58.3% vs 53.7%, X^2^ = 0.484, *p* = 0.487). Multivariate logistic regression analysis showed that non-obese NAFLD was associated with an increase in E/A < 1 (OR = 6.562, 95%CI 2.014, 21.373, *p* = 0.002).

**Conclusions:**

Non-obese NAFLD was associated with decrease of E/A, while more research will be necessary to evaluate risk of non-obese NAFLD for LV diastolic dysfunction in future.

## Introduction

In recent years, nonalcoholic fatty liver disease (NAFLD) has become the most common chronic liver disease globally. The disease is associated with many metabolic risk factors such as obesity, diabetes mellitus (DM), insulin resistance, dyslipidemia [1]. In the general population, the prevalence of NAFLD is estimated to be 25% in the world [2] and it is about 30% in China [3]. Previous studies have shown that NAFLD is closely related to obesity which will increase the prevalence of NAFLD [4], while it may also affect normal-weight individuals, a condition termed as non-obese NAFLD [5], which includes individuals with BMI < 30 kg/m^2^ in the Caucasian population and BMI < 25 kg/m^2^ in the Asian population [6, 7]. The global incidence of NAFLD in the non-obese population was about 25 per 1000 person-years and around 40% of the global NAFLD population were classified as non-obese [5, 8]. In Asia, about 30% of NAFLD population were non-obese [9]. The prevalence of NAFLD is about 7–19% [10, 11] and 8–20% among people with BMI < 25 kg/m^2^ in Asia and China respectively [12, 13], which is increasing with years [14].

Non-obese NAFLD is similar to obese NAFLD in pathophysiological mechanisms, such as hepatic lipid accumulation [15], insulin resistance [13], metabolic dysfunction of visceral fat [16], genetic susceptibility [17]. Many studies have shown that NAFLD has an adverse impact on the cardiac structure and function [18–20], which may associate with myocardial glucose uptake, myocardial fat infiltration, inflammation, oxidative stress [21, 22]. Studies have shown that an incrementally increased risk for left ventricular (LV) diastolic dysfunction according to fibrosis grade was prominent in the non-obese population [23]. However, less studies are about the effect of non-obese NAFLD on LV structure and function, so the purpose of this study is to assess the correlation of non-obese NAFLD with LV diastolic dysfunction by comparing echocardiographic parameters of LV between non-obese NAFLD group and control group.

## Methods

### Subjects

The subjects of this cross-sectional study were inpatients from the Department of Geriatrics, Peking University People's Hospital from January 2018 to December 2020. All inpatients were hospitalized for physical examination. The inclusion criteria were: (1) age ≥ 40 years old; (2) BMI < 25 kg/m^2^; (3) the imaging examination of liver (abdominal ultrasound or CT) and echocardiography were performed during hospitalization; (4) complete demographic, laboratory and imaging information. The exclusion criteria refer to the Guidelines of Prevention and Treatment for Nonalcoholic Fatty Liver Disease (2018 Updated Edition) [24] as follows: (1) excessive alcohol intake (> 30 g/d in men and > 20 g/d in women); (2) detected positive serum markers of hepatitis B and C; (3) secondary causes of fatty liver including viral hepatitis, drug-induced liver disease, autoimmune liver disease, hepatolenticular degeneration, total parenteral nutrition, inflammatory bowel disease, celiac disease, Cushing's syndrome, β-lipoprotein deficiency, lipid atrophy diabetes mellitus, Mauriac syndrome; (4) end-stage liver diseases including hepatic fibrosis, liver cirrhosis, liver cancer and liver failure; (5) basic heart diseases including coronary heart disease, congenital heart disease, valvular heart disease, pulmonary heart disease, hypertrophic cardiomyopathy, cardiac surgery, aortic dissection, heart failure; (6) chronic renal failure or malignancies; (7) pregnancy. Patients who met the inclusion criteria were divided into non-obese NAFLD group and control group according to the results of imaging examination of liver. The imaging diagnostic criteria of NAFLD refer to the Guidelines for Management of Nonalcoholic Fatty Liver Disease (2010 Revision) [25].

### Clinical and laboratory evaluations

Complete blood count, blood biochemistry, blood glucose metabolism and other indicators of patients were collected retrospectively. Alanine aminotransferase (ALT), aspartate aminotransferase (AST), γ-glutamyl transpeptidase (γ-GT), serum albumin (Alb), serum creatinine (Cr), serum uric acid (UA), fasting blood glucose (FBG), total cholesterol (TC), triglyceride (TG), high-density lipoprotein cholesterol (HDL-C) and low-density lipoprotein cholesterol (LDL-C) were detected by automatic biochemical analyzer AU5832. Hemoglobin (Hb) and platelet count (PLT) were measured by blood cell analyzer DxH800, HbA1c was measured by glycosylated hemoglobin analyzer Primus9210, and estimated glomerular filtration rate (eGFR) was obtained by CKD-EPI method [26]. Height, body weight, heart rates (HR), systolic blood pressure (SBP) and diastolic blood pressure (DBP) were measured using a digital scale. The body mass index (BMI) was calculated as weight (kg)/height (m^2^). The body surface area (BSA, male) was calculated as 0.0057 × height (cm) + 0.0121 × weight (kg) + 0.0082, and BSA (female) was calculated as 0.0073 × height (cm) + 0.0127 × weight (kg)-0.2106 [27]. Obesity is defined as BMI ≥ 25 kg/m^2^ and non-obesity is defined as BMI < 25 kg/m^2^ [7]. We collected current comorbidities, including hypertension, DM and obstructive sleep apnea–hypopnea syndrome (OSAHS), and medication history, including antihypertensive, lipid-lowering and hypoglycemic drugs. Smoking history and family history of heart diseases were also collected. Smokers were defined as individuals who had a continuous or cumulative smoking time ≥ 1 year.

### Echocardiographic evaluations

The results of echocardiography were collected retrospectively. Echocardiography was performed by Acusonsc-2000 Full Digital Color Doppler Ultrasonic Instrument, which was completed and reviewed by two sonographers (at least one of them is associate chief physician or chief physician). In this study, the results of echocardiography were used to evaluate the cardiac structure and function, and the echocardiographic parameters included interventricular septal thickness at diastole (IVSd), left ventricular posterior wall thickness at diastole (LVPWd), left ventricular mass (LVM), left ventricular mass index (LVMI), ejection fraction (EF), left ventricular end-systolic diameter (LVESD), left ventricular end-diastolic diameter (LVEDD), the peak velocity of the early filling (E) wave, the peak velocity of the atrial contraction (A) wave and E/A. E/A ratio is usually > 1 in healthy adults while it often decreases with aging and is affected by HR. In this study, LV diastolic function was assessed using E/A ratio and E value. Normal LV diastolic function was defined as 0.8 < E/A < 2. LV diastolic dysfunction was then graded as 1 (mild), 2 (moderate), and 3 (severe). Grade 1 diastolic dysfunction, which is also called delayed relaxation filling pattern, was defined as 1) E/A ≤ 0.8 and 2) E ≤ 50 cm/s. Grade 2 diastolic dysfunction, which is called pseudonormal filling pattern, was defined as 1) E/A ≤ 0.8 and 2) E > 50 cm/s. Grade 3, which is called restrictive filling pattern, was defined as E/A ≥ 2 [28].

### Statistical analysis

Statistical analyses were conducted using SPSS 26.0 software package of IBM. The measurement data conforming to the normal distribution was expressed as the mean ± standard deviation (‾x ± s), and the independent sample t-test or variance analysis was used to compare the continuous variables between two groups. The categorical variables were analyzed by x^2^ test. Univariate logistic regression was used to find out confounding factors, and two-class logistic regression (backward: LR) was used to analyze the relationship between non-obese NAFLD and LV diastolic dysfunction. *p* value of < 0.05 was considered statistically significant.

## Results

### General data

A total of 316 subjects met the inclusion criteria for the study and were finally included in this analysis, which including 118 males and 198 females, with an average age of (69 ± 12) years, (72 ± 13) years for males and (67 ± 12) years for females respectively. According to the imaging results, 72 subjects (22.8%) were diagnosed as non-obese NAFLD and 244 subjects (77.2%) belonged to control group. The general data of the subjects with and without non-obese NAFLD is provided in Table 1. BMI and BSA were significantly higher in subjects with non-obese NAFLD compared with control group (*p* < 0.01), but there was no significant statistical difference in sex composition, age, HR, SBP and DBP between the two groups.

**Table 1:** Comparison of general data between non-obese NAFLD group and control group

	**Non-obese NAFLD** **group(*n* = 72)**	**Control group** **(*n* = 244)**	***t/x***^***2***^	***P-value***
Males〔n(%)〕	22(30.6)	96(39.3)	1.835	0.176
Age(years)	67.57 ± 11.59	69.62 ± 12.88	1.287	0.200
BMI(kg/m^2^)	22.82 ± 1.74	21.85 ± 2.26	-3.896	** < 0.001**
BSA(m^2^)	1.74 ± 0.13	1.69 ± 0.13	-2.630	**0.009**
HR	73.36 ± 8.24	73.98 ± 7.44	0.600	0.549
SBP	128.13 ± 13.84	128.94 ± 16.37	0.359	0.720
DBP	75.94 ± 10.23	73.65 ± 9.34	-1.655	0.099

### Biochemical and glucose metabolism

Table 2 describes the biochemical and glucose metabolic characteristics of the study cohort according to the presence of non-obese NAFLD. Subjects with non-obese NAFLD had higher ALT, γ-GT, TG, Alb, UA, Hb, FBG and HbA1c, and lower HDL-C than subjects in the control group (*p* < 0.05). There was no significant difference in other indices.

**Table 2:** Comparison of biochemistry and glucose metabolism between non-obese NAFLD group and control group

	**Non-obese NAFLD** **group(*n* = 72)**	**Control group** **(*n* = 244)**	***t/x***^***2***^	***P-value***
ALT(U/L)	20.69 ± 9.63	16.29 ± 9.90	-3.331	**0.001**
AST(U/L)	20.19 ± 4.86	20.06 ± 6.96	-0.155	0.877
γ-GT(U/L)	28.31 ± 22.77	23.01 ± 18.14	-2.042	**0.042**
TC(mmol/L)	4.79 ± 1.17	4.51 ± 1.00	-1.864	0.065
TG(mmol/L)	1.88 ± 1.23	1.19 ± 0.58	-4.668	** < 0.001**
HDL-C(mmol/L)	1.17 ± 0.29	1.30 ± 0.34	2.990	**0.003**
LDL-C(mmol/L)	2.95 ± 0.85	2.73 ± 0.98	-1.680	0.094
Alb(g/L)	40.74 ± 3.83	38.82 ± 4.28	-3.412	**0.001**
UA(umol/L)	354.85 ± 81.05	304.18 ± 77.59	-3.897	** < 0.001**
Cr(umol/L)	66.31 ± 17.39	71.58 ± 22.51	1.834	0.068
eGFR(ml/min*1.73m2)	86.76 ± 15.71	83.50 ± 17.21	-1.439	0.151
Hb(g/L)	135.54 ± 12.51	127.23 ± 15.31	-4.211	** < 0.001**
PLT(× 10^9^/L)	214.13 ± 44.86	203.52 ± 67.82	-1.551	0.123
FBG(mmol/L)	6.02 ± 1.85	5.15 ± 1.13	-3.757	** < 0.001**
HbA1c(%)	6.73 ± 1.38	6.01 ± 0.93	-4.043	** < 0.001**

### Complications and medications

The subjects' complications and medications are compared in Table 3. Subjects with non-obese NAFLD had higher prevalence rates of DM and longer course of DM than subjects in the control group (*p* < 0.05). The proportion of patients taking hypoglycemic drugs in the non-obese NAFLD group was significantly higher than that in the control group (*p* < 0.01).

**Table 3:** Comparison of complications and medications between non-obese NAFLD group and control group

	**Non-obese NAFLD** **group(*n* = 72)**	**Control group** **(*n* = 244)**	***t/x***^***2***^	***P-value***
Hypertension〔n(%)〕	35(50.0)	102(42.1)	1.359	0.244
Course of hypertension(years)	6.85 ± 10.82	6.48 ± 11.17	-0.248	0.804
DM〔n(%)〕	30(42.9)	52(21.6)	12.653	** < 0.001**
Course of DM(years)	4.96 ± 7.66	2.52 ± 5.89	-2.461	**0.016**
OSAHS〔n(%)〕	0(0)	2(0.9)	0.000	1.000
Smoking history〔n(%)〕	8(11.1)	32(13.1)	0.202	0.653
Family history of heart disease〔n(%)〕	7(9.7)	8(3.3)	3.779	0.052
History of taking lipid-lowering drugs〔n(%)〕	23(31.9)	67(27.5)	0.549	0.459
History of taking antihypertensive drugs〔n(%)〕	32(44.4)	95(38.9)	0.702	0.402
History of taking hypoglycemic drugs〔n(%)〕	30(41.7)	51(20.9)	12.575	** < 0.001**

### LV structure and function

LV structure and function of two groups were assessed by subjects' echocardiographic parameters, which are compared in Table 4. Subjects with non-obese NAFLD had more unfavorable echocardiographic parameters, including a lower E/A and a lower LVEDD, than the control group (*p* < 0.05). There was no significant difference in other indices.

**Table 4:** Comparison of echocardiographic characteristics between non-obese NAFLD group and control group

	**Non-obese NAFLD** **group(*n* = 72)**	**Control group** **(*n* = 244)**	***t***	***P-value***
IVSd(cm)	0.91 ± 0.15	0.88 ± 0.13	-1.628	0.107
LVPWd(cm)	0.84 ± 0.12	0.87 ± 0.24	0.296	0.397
LVM(g)	130.14 ± 28.95	132.94 ± 34.91	0.684	0.495
LVMI(g/m2)	74.82 ± 15.26	78.44 ± 19.68	1.644	0.102
EF(%)	68.49 ± 5.21	69.64 ± 5.55	1.563	0.119
LVESD(cm)	2.78 ± 0.31	2.81 ± 0.33	0.716	0.474
LVEDD(cm)	4.51 ± 0.42	4.64 ± 0.43	2.182	**0.030**
E(cm/s)	68.34 ± 15.94	72.26 ± 18.05	1.664	0.097
A(cm/s)	88.16 ± 16.87	86.05 ± 20.66	-0.789	0.431
E/A	0.80 ± 0.22	0.88 ± 0.35	2.528	**0.012**

In the non-obese NAFLD group, 42 (58.3%) subjects had LV diastolic disfunction, including 8 (11.1%) subjects presented with grade 1 and 34 (47.2%) subjects presented with grade 2. In the control group, 131 (53.7%) subjects had LV diastolic disfunction, including 29 (11.9%) subjects presented with grade 1, 98 (40.2%) subjects presented with grade 2 and 4 (1.6%) subjects presented with grade 3. The prevalence rates of LV diastolic dysfunction in two groups were similar (58.3% vs 53.7%, X^2^ = 0.484, *p* = 0.487). E/A ratio is usually > 1 in healthy adults despite the lack of diagnostic significance, then we also analyzed the proportions of E/A < 1. The proportion of patients with E/A < 1 in the non-obese NAFLD group was significantly higher than the control group (*p* < 0.05). The specific data are shown in Table 5.

**Table 5:** Comparison of LV diastolic dysfunction between non-obese NAFLD group and control group

	**Non-obese NAFLD** **group(*n* = 72)**	**Control group** **(*n* = 244)**	***X***^***2***^	***P-value***
LV diastolic disfunction 〔n(%)〕	42(58.3)	131(53.7)	0.484	0.487
Grade 1〔n(%)〕	8(11.1)	29(11.9)	0.032	0.858
Grade 2〔n(%)〕	34(47.2)	98(40.2)	1.139	0.286
Grade 3〔n(%)〕	0(0)	4(1.6)	0.244	0.622
E/A < 1〔n(%)〕	60(83.3)	168(68.9)	5.802	**0.016**

### Non-obese NAFLD and LV diastolic dysfunction

Although there is no statistical difference in LV diastolic dysfunction proportions of two groups, the non-obese NAFLD group had a lower E/A and a higher proportion of E/A < 1, maybe suggesting worse LV diastolic function, so we analyzed the association between non-obese NAFLD and LV diastolic dysfunction using logistic regression analysis. According to whether 0.8 < E/A < 2, 173 subjects (54.7%) were divided into LV diastolic dysfunction group (abnormal group) and 143 subjects (45.3%) belonged to normal group. There were 42 (24.3%) and 30 (21.0%) non-obese NAFLD patients in the abnormal group and the normal group respectively. With the LV diastolic dysfunction as dependent variable, univariate logistic regression analysis showed that age, BMI, hypertension, course of hypertension, course of DM, history of taking antihypertensive drugs, HR, SBP, HbA1c, Alb and eGFR were associated with LV diastolic dysfunction (*p* < 0.05). In the univariate model, subjects with non-obese NAFLD had a 1.2-fold increased risk for LV diastolic dysfunction with no statistical significance (OR = 1.208, 95%CI 0.710, 2.055, *p* = 0.487, Table 6).

**Table 6:** Univariate analysis of the risk of left ventricular diastolic dysfunction

	***β***	***SE***	***Wald***	***OR***	***95%CI***		***P-value***
					***Lower***	***Upper***	
Male	0.352	0.236	2.228	1.422	0.896	2.257	0.136
Age	0.097	0.012	62.94	1.101	1.075	1.128	** < 0.001**
BMI	0.145	0.054	7.331	1.156	1.041	1.284	**0.007**
BSA	-1.076	0.902	1.422	0.341	0.058	1.998	0.233
Hypertension	0.966	0.238	16.498	2.628	1.649	4.190	** < 0.001**
Course of hypertension	0.047	0.013	13.189	1.048	1.022	1.075	** < 0.001**
DM	0.402	0.263	2.326	1.494	0.892	2.504	0.127
Course of DM	0.041	0.019	4.520	1.042	1.003	1.082	**0.034**
OSAHS	-0.223	1.419	0.025	0.800	0.050	12.919	0.875
NAFLD	0.189	0.271	0.483	1.208	0.710	2.055	0.487
History of taking lipid-lowering drugs	0.299	0.253	1.397	1.349	0.821	2.215	0.237
History of taking antihypertensive drugs	0.900	0.239	14.127	2.459	1.538	3.932	** < 0.001**
History of taking hypoglycemic drugs	0.454	0.265	2.945	1.575	0.938	2.645	0.086
Smoking history	-0.218	0.339	0.415	0.804	0.414	1.561	0.519
Family history of heart disease	-0.060	0.530	0.013	0.942	0.333	2.664	0.942
HR	0.047	0.016	8.993	1.048	1.016	1.081	**0.003**
SBP	0.020	0.008	5.555	1.020	1.003	1.037	**0.018**
DBP	0.005	0.013	0.170	1.005	0.980	1.031	0.680
FBG	0.114	0.087	1.718	1.121	0.945	1.329	0.190
HbA1c	0.360	0.131	7.590	1.433	1.109	1.851	**0.006**
BNP	0.003	0.002	2.049	1.003	0.999	1.007	0.152
ALT	-0.006	0.011	0.282	0.994	0.972	1.016	0.596
AST	0.021	0.018	1.281	1.021	0.985	1.058	0.258
γ-GT	0.006	0.006	0.851	1.006	0.994	1.018	0.356
TC	-0.209	0.111	3.542	0.812	0.653	1.009	0.060
TG	-0.048	0.137	0.122	0.953	0.730	1.246	0.727
HDL-C	-0.337	0.339	0.988	0.714	0.367	1.388	0.320
LDL-C	-0.088	0.120	0.539	0.915	0.723	1.159	0.463
Alb	-0.082	0.031	6.914	0.921	0.867	0.979	**0.009**
UA	0.001	0.002	0.675	1.001	0.998	1.005	0.411
Cr	0.010	0.006	2.774	1.010	0.998	1.021	0.096
eGFR	-0.036	0.008	21.332	0.965	0.950	0.979	** < 0.001**
Hb	-0.005	0.008	0.441	0.995	0.980	1.010	0.507
PLT	0.002	0.002	1.230	1.002	0.998	1.006	0.267

Further stepwise multivariate logistic regression analysis, including the above-mentioned significant variables, NAFLD and DM as a known risk factor, showed that non-obese NAFLD was associated with an increase in LV diastolic dysfunction with no statistical significance (OR = 1.206, 95%CI 0.566, 2.569, *p* = 0.628, Table 7). LV diastolic dysfunction also related to age, BMI and HR (*p* < 0.001).

**Table 7:** Multivariate logistic regression analysis of the risk of left ventricular diastolic dysfunction

	***B***	***SE***	***Wald***	***OR***	***95%CI***		***P-value***
					***Lower***	***Upper***	
Age	0.118	0.017	49.559	1.125	1.089	1.163	** < 0.001**
BMI	0.313	0.086	13.281	1.368	1.156	1.619	** < 0.001**
HR	0.077	0.021	13.015	1.080	1.036	1.126	** < 0.001**
NAFLD	0.187	0.386	0.235	1.206	0.566	2.569	0.628
constant	-20.543	3.238	40.244	< 0.001	-	-	** < 0.001**

### Non-obese NAFLD and decrease of E/A

As the non-obese NAFLD group had a higher proportion of E/A < 1 than the control group, then according to whether E/A < 1, 228 subjects (72.2%) were divided into decreased E/A group and 88 subjects (27.8%) belonged to normal E/A group. There were 60 (26.3%) and 12 (13.6%) non-obese NAFLD patients in the decreased E/A group and the normal E/A group respectively. With the E/A < 1 as dependent variable, univariate logistic regression analysis showed that NAFLD, gender, age, BMI, hypertension, course of hypertension, course of DM, history of taking lipid-lowering and antihypertensive drugs, SBP, HbA1c, UA, Cr and eGFR were associated with E/A < 1 (*p* < 0.05). In the univariate model, subjects with non-obese NAFLD had a 2.3-fold increased risk for E/A < 1 (OR = 2.262, 95%CI 1.150, 4.449, *p* = 0.018, Table 8).

**Table 8:** Univariate analysis of the risk of E/A < 1

	***β***	***SE***	***Wald***	***OR***	***95%CI***		***P-value***
					***Lower***	***Upper***	
Male	0.626	0.275	5.203	1.871	1.092	3.204	**0.023**
Age	0.091	0.013	48.668	1.095	1.067	1.123	** < 0.001**
BMI	0.155	0.056	7.697	1.168	1.047	1.303	**0.006**
BSA	-0.134	0.994	0.018	0.874	0.125	6.134	0.892
Hypertension	1.025	0.278	13.644	2.788	1.618	4.804	** < 0.001**
Course of hypertension	0.048	0.016	8.341	1.049	1.015	1.083	**0.004**
DM	0.576	0.314	3.372	1.779	0.962	3.290	0.066
Course of DM	0.063	0.026	5.718	1.065	1.011	1.122	**0.017**
OSAHS	-1.019	1.421	0.514	0.361	0.022	5.845	0.361
NAFLD	0.816	0.345	5.593	2.262	1.150	4.449	**0.018**
History of taking lipid-lowering drugs	0.972	0.324	9.023	2.643	1.403	4.983	**0.003**
History of taking antihypertensive drugs	1.101	0.287	14.721	3.007	1.713	5.276	** < 0.001**
History of taking hypoglycemic drugs	0.585	0.313	3.492	1.794	0.972	3.313	0.062
Smoking history	-0.255	0.364	0.491	0.775	0.380	1.581	0.483
Family history of heart disease	0.063	0.598	0.011	1.065	0.330	3.436	0.917
HR	0.026	0.017	2.360	1.026	0.993	1.060	0.124
SBP	0.030	0.010	8.953	1.030	1.010	1.050	**0.003**
DBP	0.014	0.015	0.857	1.014	0.985	1.043	0.355
FBG	0.102	0.101	1.035	1.108	0.910	1.349	0.309
HbA1c	0.362	0.161	5.078	1.437	1.048	1.969	**0.024**
BNP	0.001	0.002	0.356	1.001	0.997	1.006	0.551
ALT	-0.003	0.012	0.062	0.996	0.973	1.021	0.457
AST	0.006	0.020	0.093	0.999	0.968	1.046	0.940
γ-GT	-0.007	0.006	1.129	0.998	0.982	1.006	0.309
TC	-0.186	0.122	2.295	0.831	0.653	1.056	0.130
TG	0.172	0.179	0.916	1.187	0.835	1.687	0.338
HDL-C	-0.846	0.375	5.099	1.018	0.206	0.894	0.672
LDL-C	-0.052	0.129	0.159	0.950	0.737	1.224	0.690
Alb	-0.027	0.031	0.768	0.973	0.916	1.034	0.381
UA	0.005	0.002	5.326	1.005	1.001	1.009	**0.021**
Cr	0.014	0.007	3.902	1.014	1.000	1.029	**0.048**
eGFR	-0.044	0.010	20.838	0.957	0.939	0.975	** < 0.001**
Hb	0.002	0.008	0.038	1.002	0.986	1.018	0.845
PLT	0.000	0.002	0.061	1.000	0.996	1.003	0.805

Further stepwise multivariate logistic regression analysis, including the above-mentioned significant variables and DM as a known risk factor, showed that non-obese NAFLD was associated with an increase risk in E/A < 1 (OR = 6.562, 95%CI 2.014, 21.373, *p* = 0.002, Table 9).

**Table 9:** Multivariate logistic regression analysis of the risk of E/A < 1

	***B***	***SE***	***Wald***	***OR***	***95%CI***		***P-value***
					***Lower***	***Upper***	
NAFLD	1.881	0.603	9.749	6.562	2.014	21.373	**0.002**
Age	0.104	0.021	23.810	1.109	1.064	1.157	** < 0.001**
BMI	0.283	0.108	6.812	1.326	1.073	1.640	**0.009**
constant	-13.033	3.186	16.735	** < 0.001**	-	-	** < 0.001**

## Discussion

In this study, non-obese NAFLD was associated with decrease of E/A, independent of well-identified cardiovascular risk factors, while it was not an independent risk factor of LV diastolic dysfunction.

Previously, several studies have suggested that NAFLD was an independent risk factor affecting cardiac structure and function, but there are few studies on the correlation between non-obese NAFLD and LV function or structure in Chinese adults. Therefore, this paper studied the changes of LV structure and function in hospitalized non-obese NAFLD patients, and discussed the correlation between non-obese NAFLD and LV diastolic dysfunction, aiming to provide scientific evidence for clinicians to pay attention to the cardiac structure and function of non-obese NAFLD patients.

Our study showed that compared with the control group, non-obese NAFLD patients had higher BMI, BSA, levels of liver enzymes, blood lipids, proportion of DM, and worse glucose metabolism, which were consistent with previous reports. Although BMI of a non-obese NAFLD patient is within the normal range, it is still higher than that of a healthy adult and the visceral fat index is also high [13, 29]. Obesity is related to higher all-cause mortality [30], so both obese and non-obese NAFLD patients can benefit from losing weight [31]. For non-obese NAFLD patients, a 5–10% reduction in body weight through lifestyle intervention may be a reasonable target and can also benefit them a lot [32]. There are also many drugs as good candidates to cure NAFLD/NASH, and consequently to reduce the burden of cardiovascular diseases (CVD) [33].

In this study, LV structure and function were mainly evaluated by echocardiographic parameters, among which E/A ratio and E value were important indices to evaluate LV diastolic function. In the early stage of LV diastolic dysfunction, the E value declines due to the decrease of the maximum mitral blood flow velocity in the early LV diastole, which leads to E/A < 1 [34]. With the further decline of LV diastolic function, the reduction in diastolic LV filling results in an atrial residuum, which increases left atrial (LA) pressure, causing an increase of E value. Finally, the increased load imposed on the LA as a result of a poorly compliant LV may lead to decreased atrial contractile reserve, LA enlargement, and a decrease of A value. The combined effects of a rise in E value and a decrease in A value result in an increase of E/A [28].

We compared the LV structure and function indices between non-obese NAFLD group and control group, and the results showed that non-obese NAFLD patients had lower E/A while there was no significant statistical difference in proportions of LV diastolic dysfunction. The reason why we considered the result may be that decreased E/A or declined function in non-obese NAFLD patients was at an extremely early stage so that had not yet reached the diagnostic standard of LV diastolic dysfunction. Then in addition to diastolic dysfunction, we also analyzed E/A < 1 as a dependent variable. The results of this study showed that in non-obese people, subjects with NAFLD had a 6.6-fold increased risk for decrease of E/A while non-obese NAFLD was not an independent risk factor of LV diastolic dysfunction.

Maybe the above results were still controversial because there are few similar studies about non-obese NAFLD at present. A previous research showed that non-obese NAFLD patients have worse echocardiographic measurements, including IVSd, LVEDD, LVPWd, EF, LVM and E velocity [35]. Most studies about NAFLD, whether the patient was obese or not, showed that NAFLD patients had worse cardiac structure and function [21, 36–41], even related to the degree of liver fibrosis [42]. And latest research revealed that LVMI increased progressively and LV diastolic function worsened with increasing number of steatosis scores in NAFLD patients. It also showed that liver steatosis, as identified by use of biochemical scores, predicted LV hypertrophy and diastolic dysfunction independently of blood pressure and obesity, and this association was independent of the HOMA-index for LV diastolic dysfunction [43]. Then there was a research showing that metabolic dysfunction-associated fatty liver disease patients had increased interventricular septum thickness, left ventricular posterior wall thickness, LVM and LVMI, and more patients with MAFLD had LV diastolic dysfunction compared to the normal group (60.8% vs 24.6%, *p* < 0.001).

On the contrary, some studies believed that correlation between NAFLD and cardiac function remained to be confirmed because of confounding factors such as obesity [44, 45]. A previous Chinese cohort study revealed that no significant association was observed between non-obese NAFLD and incident coronary artery disease after adjusting other traditional cardiovascular risk factors [46].

Surprisingly, non-obese NAFLD patients have smaller LVEDD, which is usually found in late stage of LV diastolic dysfunction. We consider this result probably because non-obese NAFLD patients had more complications or some medication history leading to abnormal ventricular structure. Moreover, despite the exclusion of all patients with known heart disease, there may be patients with asymptomatic coronary heart disease even if all echocardiographic results showed no abnormal wall motion. Both multivariate logistic regression analyses showed that age and BMI were independent risk factors of LV diastolic dysfunction or E/A decrease, which was consistent with previous consensus that age and BMI are independent risk factors of CVD.

Non-obese NAFLD is similar to obese NAFLD in pathophysiology and non-obese NAFLD individuals seem to have an intermediate metabolic phenotype between healthy individuals and obese NAFLD patients.

A previous study based on liver biopsy showed that compared with obese NAFLD patients, non-obese NAFLD patients had lighter degree of hepatocytic steatosis, lobular inflammation and advanced liver fibrosis, and lower prevalence of NASH (54.1% vs 71.2%, *p* < 0.001) [47], and it also believed that liver fibrosis in non-obese NAFLD patients was obviously related to metabolic disorders. Another meta-analysis including 493 non-obese NAFLD patients and 2209 obese NAFLD patients compared the liver histological features between the two groups, which also showed that pathological changes of non-obese NAFLD were mild [48]. Insulin resistance is universal in NAFLD patients whether they are obese or not [12, 49, 50]. Non-obese NAFLD patients generally had higher prevalence of DM and glucose intolerance than healthy subjects, while there was no statistical difference between non-obese and obese NAFLD patients [51]. The changes of intestinal microbiota are also associated with the progress of NAFLD and liver fibrosis [52, 53]. A previous report about gut microbiota composition showed that *Eubacterium* abundance was significantly decreased in non-obese NAFLD patients compared with that in obese NAFLD patients and healthy subjects, then the results demonstrated a negative correlation between *Eubacterium* and hepatic fibrosis and that the decrease in the abundance of *Eubacterium* producing butyric acid may play an important role in the development of non-obese NAFLD [54]. It was found that a variety of gene sites are related to the risk, disease severity, hepatic steatosis and advanced fibrosis of NAFLD, including *PNPLA3*, *TM6SF2*, *GCKR*, *MBOAT7*, *APOC3*, *HSD17B13*, etc. [55–60]. Among them, *PNPLA3* is one of the earliest genes related to NAFLD in genome-wide association studies. the *PNPLA3* rs738409 GG genotype was found in 13–19% of the general population in Asian, which is higher than that in other regions [12]. *PNPLA3* not only plays a role in increasing the susceptibility to NAFLD, but also is related to abdominal visceral fat accumulation [61], and this gene has been proved to be one of the risk factors for NAFLD in non-obese people [62]. *TM6SF2* has a protective effect on cardiovascular system, but it participates in hepatic steatosis and increases the susceptibility to NASH and hepatic fibrosis [63]. Compared with obese NAFLD patients, *TM6SF2* E167K mutation is more common in non-obese NAFLD patients [64]. A new study found that loss of immunity-related GTPase GM4951 leads to nonalcoholic fatty liver disease without obesity [65]. At present, it is considered that NAFLD is not only related to systemic insulin resistance, but also related to endothelial dysfunction, oxidative stress, plaque formation, vascular tone change, systemic inflammatory response, metabolic disorders of blood lipid and so on [66–68]. Previous studies have shown that compared with healthy people, non-obese NAFLD patients also have a higher risk of coronary heart disease [69], and there is no statistical difference between non-obese NAFLD patients and obese NAFLD patients in the risk of CVD and malignant tumors, and they all have a higher risk of all-cause mortality [65]. The main causes of death of non-obese NAFLD patients are malignant tumors and CVD [70].

Despite the fact that NAFLD is usually associated with obesity, it has also been noted that the prevalence of NAFLD is increasing in non-obese individuals. Non-obese NAFLD is similar to the obese NAFLD in pathophysiological mechanism and influence on other related diseases. Compared with the healthy individuals, the non-obese NAFLD patients have a higher risk of liver cirrhosis, hypertension, DM, coronary heart disease and other diseases, as well as the risk of all-cause death, which needs to be confirmed by more studies in the future.

The research is meaningful for clinicians and patients because it can remind clinicians to pay more attention to cardiac structure and function of non-obese NAFLD patients, and early intervention on non-obese NAFLD to delay its progress may be helpful to prevent myocardial dysfunction.

However, this study has several limitations. First, the cross-sectional design of this study was difficult to explore the causal relationship between NAFLD and LV diastolic dysfunction. Second, imaging examination were used to diagnose NAFLD and we were unable to obtain liver histological samples, the gold standard for the diagnosis of NAFLD. Third, data on visceral adiposity, such as waist circumference, was lacking. Forth, despite the exclusion of all patients with known heart disease, the further research may need the coronary angiography as the diagnostic gold standard to exclude patients with asymptomatic coronary heart disease. Fifth,we used E/A and E value to evaluate LV diastolic dysfunction, while only this approach is difficult to identify patients with Grade 2 diastolic dysfunction (pseudonormal filling pattern) when E/A is in the normal range [28]. Therefore, more accurate methods will be needed in the future. Sixth, the cohort in this research was a selected population, so may not be representative of the general population. In addition, this study only included subjects of East Asian ethnicity, so the conclusions may not be generalizable to other ethnic groups. Further studies are needed to validate our results.

In conclusion, non-obese NAFLD was associated with decrease of E/A, while more research will be necessary to evaluate risk of non-obese NAFLD for LV diastolic dysfunction in future.

## Data Availability

The datasets used and analyzed during the current study are not publicly available due not all of the researchers wish to share the data with public at present, but available from the corresponding author on reasonable request.

## Abbreviations

NAFLD: Non-alcoholic fatty liver disease
LV: Left ventricle
DM: Diabetes mellitus
ALT: Alanine aminotransferase
AST: Aspartate aminotransferase
γ-GT: γ-Glutamyl transpeptidase
Alb: Serum albumin
Cr: Serum creatinine
UA: Serum uric acid
FBG: Fasting blood glucose
TC: Total cholesterol
TG: Triglyceride
HDL-C: High-density lipoprotein cholesterol
LDL-C: Low-density lipoprotein cholesterol
Hb: Hemoglobin
PLT: Platelet count
eGFR: Estimated glomerular filtration rate
HR: Heart rates
SBP: Systolic blood pressure
DBP: Diastolic blood pressure
BMI: Body mass index
BSA: Body surface area
OSAHS: Obstructive sleep apnea–hypopnea syndrome
IVSd: Interventricular septal thickness at diastole
LVPWd: Left ventricular posterior wall thickness at diastole
LVM: Left ventricular mass
LVMI: Left ventricular mass index
EF: Ejection fraction
LVESD: Left ventricular end-systolic diameter
LVEDD: Left ventricular end-diastolic diameter
E: The peak velocity of the early filling wave
A: The peak velocity of the atrial contraction wave
BNP: Brain Natriuretic Peptide
CVD: Cardiovascular diseases
LA: Left atrial
